# Characterization of the complete chloroplast genome of *Boschniakia himalaica* J. D. Hooker & Thomson (Orobanchaceae), a medicinal species in southwest China

**DOI:** 10.1080/23802359.2019.1664952

**Published:** 2019-09-19

**Authors:** Nong Zhou

**Affiliations:** a College of Food and Biology Engineering, Chongqing Three Gorges University, Chongqing, China;; b The Chongqing Engineering Laboratory for Green Cultivation and Deep Processing of the Three Gorges Reservoir Area’s Medicinal Herbs, Chongqing Three Gorges University, Chongqing, China

**Keywords:** *Boschniakia himalaica*, chloroplast, Illumina sequencing, phylogeny

## Abstract

*Boschniakia himalaica* is a medicinal plant in southwest China. In this study, we sequenced complete chloroplast (cp) genome of *B. himalaica* to investigate its phylogenetic relationship in Orobanchaceae. The chloroplast genome was 106,466 bp in length with 37.3% overall GC content, including a large single copy (LSC) region of 49,606 bp, a small single copy (SSC) region of 5572 bp, and a pair of inverted repeats (IRs) of 25,644 bp. The cp genome contained 84 genes, including 50 protein-coding genes, 30 tRNA genes, and 4 rRNA genes. The phylogenetic analysis indicated *B. himalaica* was closely related to *Cistanche deserticola.*

Orobanchaceae is a special family of angiosperm in which most of the species have no chlorophyll. *Boschniakia* is a small genus in the family that only includes three species all over the world. In this genus, only two species can be found in China, namely *Boschniakia himalaica* J. D. Hooker & Thomson and *Boschniakia rossica* (Cham. & Schlecht.) Fedtsch. (Zhang and Nikolai 1998). Among them, *B. himalaica* is mainly distributed in Yunnan, Tibet, Shanxi, Sichuan, and Hubei provinces of China as a parasitic plant growing on root of plants of genus *Rhododendron* (Ericaceae) (Zhang et al. 2013). Plants of this species have been widely used in folk medicine in southwest China for regulating vital energy, alleviating pain, relieving cough and reducing sputum (Nanjing University of Chinese Medicine 2014). However, for such a medicinal plant, few studies have been conducted in molecular biology besides describing its chemical compositions, to this day (Wan et al. 2012; Zhang et al. 2013, 2016). Here, we reported chloroplast (cp) genome sequence of *B. himalaica* and revealed its internal relationships with other taxa in the family Orobanchaceae.

Fresh and clean leaf materials of *B. himalaica* were collected from Dali county, Yunnan, China (N 25.87°, E 100.01°), and the plant materials and a voucher specimen (No. ZDQ17024) were deposited at Dali University. Total genomic DNA was extracted using the improved CTAB method (Doyle 1987; Yang et al. 2014), and sequenced with Illumina Hiseq 2500 (Novogene, Tianjing, China) platform with pair-end (2 × 300 bp) library. About 3.64 Gb of raw reads with 12,121,830 paired-end reads were obtained from high-throughput sequencing. The raw data was filtered using Trimmomatic v.0.32 with default settings (Bolger et al. 2014). Then paired-end reads of clean data were assembled into circular contigs using GetOrganelle.py (Jin et al. 2018). Finally, the cpDNA was annotated by the Dual Organellar Genome Annotator (DOGMA; http://dogma.ccbb.utexas.edu/) (Wyman et al. 2004) and tRNAscan-SE (Lowe and Chan 2016).

The annotated cp genome was submitted to the GenBank under the accession number xxx. The total length of the cp genome was 106,466 bp, with 37.3% overall GC content. With typical quadripartite structure, a pair of IRs (inverted repeats) of 25,644 bp was separated by a small single copy (SSC) region of 5572 bp and a large single copy (LSC) region of 49,606 bp. The cp genome contained 84 genes, including 50 protein-coding genes, 30 tRNA genes, and 4 rRNA genes. Of these, 19 genes were duplicated in the inverted repeat regions, 6 genes, and 6 tRNA genes contained one intron, while one gene (*clpP*) had two introns.

To investigate its taxonomic status, a total of 15 cp genome sequences of Orobanchaceae species were downloaded from the NCBI database used for phylogenetic analysis. After using MAFFT V.7.149 for aligning (Katoh and Standley 2013), jModelTest v.2.1.7 (Darriba et al. 2012) was used to determine the best-fitting model for the cp genomes. Then Bayesian inference (BI) was performed by MrBayes v.3.2.6 (Ronquist et al. 2012) with *Solanum lycopersicu* (No. AC_000188) as outgroup. The results showed that *B. himalaica* was closely related to *Cistanche deserticola* (Figure 1), the original species of famous traditional Chinese medicine Cistanches Herba (Chinese Pharmacopoeia Committee 2015). This study would afford scientific evidence for resource development of *B. himalaica* and further phylogeny.

**Figure 1.**
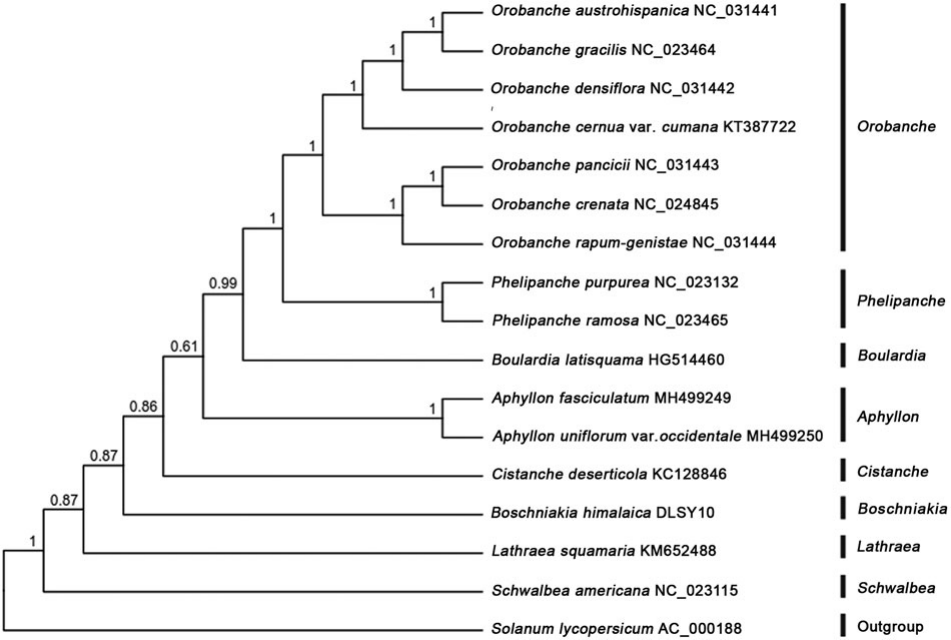
Bayesian inference (BI) tree of 16 species within the family Orobanchaceae based on the complete plastome sequences using *Solanum lycopersicu* (No. AC_000188) as outgroup.
